# Attitudes and perceptions of Chinese oncologists towards artificial intelligence in healthcare: a cross-sectional survey

**DOI:** 10.3389/fdgth.2024.1371302

**Published:** 2024-09-03

**Authors:** Ming Li, Xiaomin Xiong, Bo Xu

**Affiliations:** ^1^ Department of Health Policy Management, Bloomberg School of Public Health, Johns Hopkins University, Baltimore, MD, United States; ^2^ Department of Breast Oncology, Chongqing University Cancer Hospital and Chongqing University School of Medicine, Institute of Intelligent Oncology, Chongqing University, Chongqing, China; ^3^ Key Laboratory of Breast Cancer Prevention and Therapy, Department of Biochemistry and Molecular Biology, Ministry of Education, National Cancer Research Center, Tianjin Medical University Cancer Institute and Hospital, Tianjin, China

**Keywords:** artificial intelligence, AI, oncologists, attitude, perception

## Abstract

**Background:**

Artificial intelligence (AI) is transforming healthcare, yet little is known about Chinese oncologists’ attitudes towards AI. This study investigated oncologists’ knowledge, perceptions, and acceptance of AI in China.

**Methods:**

A cross-sectional online survey was conducted among 228 oncologists across China. The survey examined demographics, AI exposure, knowledge and attitudes using 5-point Likert scales, and factors influencing AI adoption. Data were analyzed using descriptive statistics and chi-square tests.

**Results:**

Respondents showed moderate understanding of AI concepts (mean 3.39/5), with higher knowledge among younger oncologists. Only 12.8% used ChatGPT. Most (74.13%) agreed AI is beneficial and could innovate healthcare, 52.19% respondents expressed trust in AI technology. Acceptance was cautiously optimistic (mean 3.57/5). Younger respondents (∼30) show significantly higher trust (*p* = 0.004) and acceptance (*p* = 0.009) of AI compared to older respondents, while trust is significantly higher among those with master’s or doctorate vs. bachelor’s degrees (*p* = 0.032), and acceptance is higher for those with prior IT experience (*p* = 0.035).Key drivers for AI adoption were improving efficiency (85.09%), quality (85.53%), reducing errors (84.65%), and enabling new approaches (73.25%).

**Conclusions:**

Chinese oncologists are open to healthcare AI but remain prudently optimistic given limitations. Targeted education, especially for older oncologists, can facilitate AI implementation. AI is largely welcomed for its potential to augment human roles in enhancing efficiency, quality, safety, and innovations in oncology practice.

## 1 Introduction

Over the past few years, Artificial Intelligence (AI) has the potential to revolutionize the landscape of medicine and healthcare (1). Artificial Intelligence (AI), with its various technologies such as Machine Learning (ML), Deep Learning, Natural Language Processing (NLP), Robotic Process Automation (RPA), Large language models (LLMs) is transforming the medical practice and healthcare delivery (2, 3).

AI applications are expanding into areas previously exclusive to human experts. AI is revolutionizing healthcare by automated analysis of medical images, predictive risk models, optimized treatment plans, virtual nursing assistants, and intelligent hospital workflow management, thus improving the patient experience (4). Despite these advancements, the full potential of AI in healthcare remains untapped.

AI is transforming cancer care and research through expanded applications of detection, diagnosis, tumor classification, treatment optimization, drug development, and outcome prediction (5–7). AI shows significant promise for genomic tumor characterization, personalized medicine, radiotherapy, accelerating clinical trials, and bridging the gap from Research to Practice (8). As AI integration expands, it could enable more accurate and effective cancer care (9). Overall, AI offers immense opportunities to revolutionize oncology through enhanced analytics and personalized insights.

AI holds tremendous potential to enhance patient outcomes and reshape medicine, but responsible application is crucial. Ongoing advances and integration of AI into healthcare will determine how AI augments and improves clinical practice in the coming decades.

A key driver for successful implementation and uptake of high-technology systems is the perception and attitude of physicians (10, 11). Doctors need a rational attitude, and a realistic understanding of health AI applications’ potential uses and limitations, which encourage oncologists to use AI products in clinical practice actively.

AI products are now the subject of more considered discussion. However, it remains unknown what oncologists thought of AI technology. Little is known about the attitude of oncologists towards AI. To address this issue, we performed an online survey amongst conchologists to assess their perceptions and thoughts on AI in healthcare in the future, thus facilitating the adoption of AI tools that augment human expertise.

We could identify the impact factors and their thoughts about AI by exploring the oncologists’ attitudes toward AI products. These findings will help us understand the AI product acceptance process by considering the context of use. Furthermore, the research will inform executives and key decision-makers about the requirements for AI acceptance and effective use of AI in clinical settings.

## 2 Method

### 2.1 Study design

This cross-sectional investigation deployed a digital survey to probe the attitudes of oncologists towards Artificial Intelligence (AI), along with the determinants shaping its utilization within their medical practices. The questionnaire, stemming from an exhaustive literature review coupled with insights drawn from comprehensive interviews with practicing oncologists, was disseminated via the WenJuan minor program, an online survey utility.

In addition to the characteristics of doctors, the main contents of the questionnaire in these literatures include the understanding of AI knowledge, perceptions of AI benefits, performance expectancy, behavior intention, the factors influencing AI adoption, and the concerns about AI.

The survey instrument was structured into four parts, encompassing 19 questions with 28 items. The primary section gathered demographic and professional characteristics of the oncologists with 7 items. The following section probed the degree of oncologists’ exposure to AI products with 2 items. The third section with 16 items evaluated their knowledge, perceptions, and attitudes towards AI using a 5-point Likert scale, ranging from “strongly disagree” to “strongly agree,” which gauged the participants’ agreement level with statements concerning their current stance on AI. The concluding section with 2 items was structured to elucidate the factors shaping their adoption of AI. The last question is open-ended, allowing for the submission of free-text responses.

### 2.2 Study population

The scope of the study population was narrowed to oncologists practicing in Chinese hospitals, intentionally excluding general practitioners, surgeons, physicians, and medical students. The survey, anonymized and presented in Chinese, was distributed via WeChat, the country’s largest Social Networking Service platform.

The data collection process was carried out from April 4th to June 30th, 2023, post the approval from the Institutional Review Board of ChongQing University Cancer Hospital. Incomplete submissions or entries from non-target respondents, such as radiologists, were excluded from the study.

Respondents were required to confirm an electronic consent form before proceeding with the questionnaire.

### 2.3 Data analysis

Following the survey’s closure, the collected responses were downloaded as an Excel file. Descriptive statistical methods were employed to collate the survey outcomes, focusing particularly on the ranking of influential factors pertaining to the adoption of AI by oncologists.

We employed the Likert scale to capture a wide spectrum of attitudes towards AI, with survey responses ranging from 1 (strongly disagree) to 5 (strongly agree) calculate mean score and standard deviation. Cronbach’s alpha is also calculated to verify the internal consistency of the questionary.

Associations between oncologists’ attributes and their attitudes towards AI were gauged using the Chi-squared test. For ease of interpretation, the categories “strongly disagree” and “disagree” were amalgamated as disagreement, and “agree” and “strongly agree” as agreement. Variations in responses, as influenced by factors such as gender, education, and experience length, were evaluated using Pearson’s correlation. The threshold for statistical significance was set at an alpha level of 0.05, with *P*-values below 0.05 deemed statistically significant.

The data analyses were conducted utilizing the Statistical Package for the Social Sciences (SPSS) version 22.0.

## 3 Result

During the survey period, a total of 228 oncologists completed the questionnaire, meanwhile 318 oncologists accessed the online version. The average time taken to fill out the questionnaire was 5 min and 17 s.

### 3.1 Respondent demographics

Survey responses were collected from a total of 228 respondents in China. The descriptive data of respondent’s demographics are provided in Supplementary Table 1.

The sample consisted predominantly of males (*n* = 135, 59.21%) while females comprised 40.79% (*n* = 93). Age distribution revealed that a majority of the respondents were in the age range of 31–40 years (*n* = 95, 41.67%), followed by 41–50 years (*n* = 80, 35.09%). The participants in the ∼30 years (*n* = 28, 12.28%) and 51–60 years (*n* = 25, 10.96%) age categories formed the minority. In terms of clinical practice experience, a significant portion of the participants had 11–20 years of experience (*n* = 126, 55.26%), whereas those with 0–10 years (*n* = 49, 21.49%) and 21 years and above (*n* = 53, 23.25%) were relatively fewer. Regarding educational background, the highest percentage of participants held a Bachelor’s degree (*n* = 89, 39.04%), closely followed by Master’s degree holders (*n* = 83, 36.40%), with Doctorate degree holders forming the smallest group (*n* = 56, 24.56%).When exploring specialties, a considerable proportion were specialized in Medical Oncology (*n* = 97, 42.54%). This was followed by Surgical Oncology (*n* = 77, 33.77%), Radiation Therapy (*n* = 40, 17.54%), and Other specialties (*n* = 14, 6.14%).In terms of hospital type, most participants worked in University Hospitals (*n* = 148, 64.91%), as opposed to Non-University Hospitals (*n* = 80, 35.09%). In the context of IT project experience, only a small fraction of participants had previous experience (*n* = 35, 15.35%), while the vast majority indicated no experience (*n* = 193, 84.65%).

### 3.2 Exposure to AI

**Figure 1:**
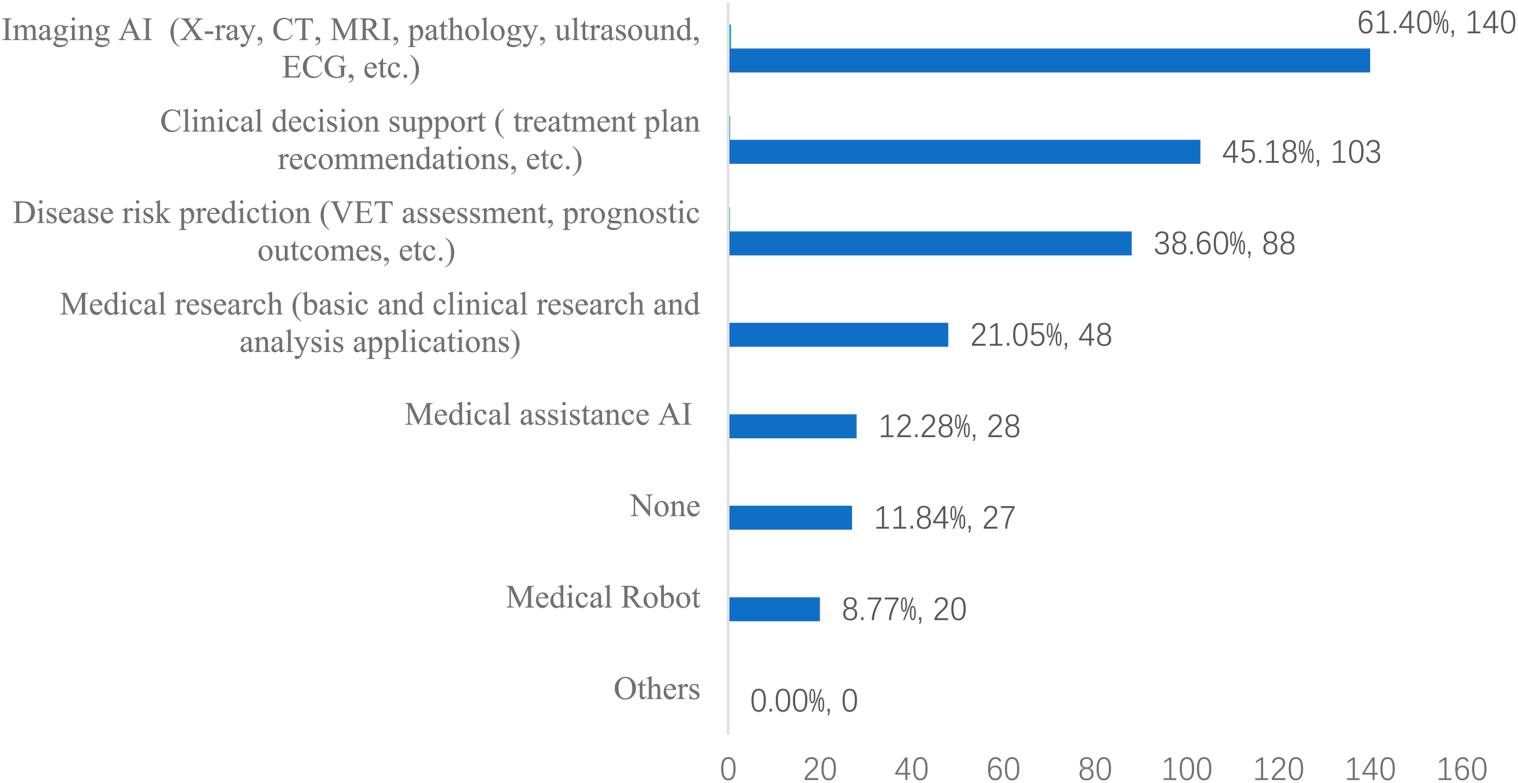
The AI products usage (*n* = 228).

The most exposed to AI product was Imaging AI, utilized in procedures such as x-ray, CT, MRI, pathology, ultrasound, and ECG, by 61.40% (*n* = 140) of the respondents. Clinical decision support tools, which include treatment plan recommendations, were also widely used, with 45.18% (*n* = 103) of participants reporting their usage.AI applications in disease risk prediction, such as VET assessment and prognostic outcomes, were used by 38.60% (*n* = 88) of participants. Medical research applications, including basic and clinical research and analysis applications, were adopted by 21.05% (*n* = 48) of the respondents. Furthermore, medical assistance AI was utilized by 12.28% (*n* = 28) of the participants, whereas a small percentage of respondents reported the use of Medical Robots (8.77%, *n* = 20). Interestingly, a portion of the participants reported no AI product usage (11.84%, *n* = 27), and no participant reported the use of other AI products (Figure 1).

### 3.3 Moderate understanding of AI and the willingness to learning

We analyzed survey data from a sample of 228 oncologists to elucidate their perceptions and understanding of Artificial Intelligence (AI), with particular focus on AI in the context of medical practice. These findings were available in Table 1.

**Table 1:** Knowledge and learning willingness on AI (*n* = 228).

Question	Strongly disagree		Disagree		Neutral		Agree		Strongly agree		SD	Variance	Cronbach's alpha
I know much about the terms “artificial intelligence”, “machine learning” and “deep learning”	16	7.02%	29	12.72%	80	35.09%	56	24.56%	47	20.61%	1.15	1.31	0.5
I know much about the limitation of AI	22	9.65%	34	14.91%	94	41.23%	43	18.86%	35	15.35%	1.15	1.33	
I am willing to learn knowledge of AI	12	5.26%	12	5.26%	51	22.37%	54	23.68%	99	43.42%	1.16	1.34	0.82
I think it is necessary to train clinicians on AI knowledge and applications	13	5.70%	7	3.07%	53	23.25%	62	27.19%	93	40.79%	1.13	1.27	

On understanding of AI-related terms such as “Artificial Intelligence”, “Machine Learning”, and “Deep Learning”, the mean score was 3.39 (SD = 1.15), indicating a moderate level of comprehension. Notably, 35.09% of the respondents had a moderate understanding (rating 3), while a combined 44.17% demonstrated high or very high understanding (rating 4 or 5 respectively).When inquired about the limitations of AI, the mean score was 3.15 (SD = 1.15), reflecting an intermediate understanding. While 41.23% of respondents expressed a moderate understanding, 34.21% demonstrated high or very high understanding, showcasing their familiarity with AI’s capabilities and constraints.

The respondents exhibited a strong personal interest in learning about AI, as shown by a mean score of 3.95 (SD = 1.16). An impressive 43.42% showed a very high willingness to learn about AI. Moreover, a considerable 40.79% strongly agreed that “doctors should undergo AI training,” reflecting a mean score of 3.94 (SD = 1.13], and underlining the recognized necessity for AI competence in contemporary medical practice.

### 3.4 General good perceptions and intention to utilize AI

The surveyed oncologists acknowledged the benefits of medical AI, with 45.18% strongly agreeing that AI is beneficial, corresponding to a mean score of 4.12 (SD = 0.98). However, their acceptance of AI technology was somewhat cautious, with a mean score of 3.57 (SD = 1.01). 19.30% strongly agreed that they trust current medical AI technology, and 29.39% were in strong agreement to its acceptance, indicating a readiness to integrate AI into practice but also an awareness of its limitations.

The proposition that AI could outperform doctors in certain areas received a varied response, with 22.81% strongly agreeing, reflected in a mean score of 3.43 (SD = 1.21).

Oncologists expressed a strong belief that AI will bring about significant changes to oncology (mean score = 3.81, SD = 1.08), the medical system (mean score = 3.87, SD = 1.07), and is a future trend in medicine (mean score = 3.9, SD = 1.1).

Regarding personal willingness to use AI, the mean score was 4.07 (SD = 1.01), with 42.11% strongly agreeing to use AI if available. These findings were available in Table 2.

**Table 2:** Oncologists’ perceptions and intention to utilize AI.

Question	Strongly disagree		Disagree		Neutral		Agree		Strongly agree		SD	Variance	Cronbach's alpha
I believe “AI is beneficial”	5	2.19%	7	3.07%	47	20.61%	66	28.95%	103	45.18%	0.98	0.96	0.77
I Trust in health AI technology	9	3.95%	16	7.02%	84	36.84%	75	32.89%	44	19.30%	1.01	1.01	
I accept AI technology	7	3.07%	13	5.70%	70	30.70%	71	31.14%	67	29.39%	1.03	1.06	
I believe “AI will bring innovation to oncology”	7	3.07%	20	8.77%	57	25.00%	69	30.26%	75	32.89%	1.08	1.17	0.85
I believe “AI will bring innovation to healthcare system”	8	3.51%	15	6.58%	54	23.68%	72	31.58%	79	34.65%	1.07	1.15	
I believe “AI is the future development trend of healthcare”	10	4.39%	12	5.26%	53	23.25%	68	29.82%	85	37.28%	1.1	1.2	
I am willing to use AI if possible	8	3.51%	6	2.63%	43	18.86%	75	32.89%	96	42.11%	1.01	1.03	0.89
if possible, I will use AI as much as possible	9	3.95%	9	3.95%	55	24.12%	71	31.14%	84	36.84%	1.06	1.12	

### 3.5 Mixed feeling about comparation AI capabilities with doctors’ capabilities

“Some aspects of current AI technology have exceeded the average ability of doctors,” the distribution of opinions is balanced, with 30.26% of respondents being neutral and 49.13% agreeing & strongly agreeing. This suggests a recognition of AI’s potential in specific medical tasks, albeit with a significant portion still holding neural position.

A small minority of respondents (4.39%) strongly disagreed with the statement “AI will not replace doctors”, paralleled by an equal percentage (4.39%) who merely disagreed. A slightly larger proportion (12.72%) expressed a neutral stance on the issue. More significantly, a substantial portion (24.56%) agreed with the statement, and an overwhelming majority (53.95%) strongly agreed that AI will not replace doctors. These findings were available in Table 3.

**Table 3:** Comparation AI capabilities with doctors’ capabilities.

Question	Strongly disagree		Disagree		Neutral		Agree		Strongly agree		SD	Variance	Cronbach's alpha
I believe “Some aspects of current AI technology have exceeded the average ability of doctors”	20	8.77%	27	11.84%	69	30.26%	60	26.32%	52	22.81%	1.21	1.46	0.64
I believe “AI will replace doctor”	53	23.25%	32	14.04%	68	29.82%	37	16.23%	38	16.67%	1.38	1.89	

### 3.6 Statistically significant associations

Regarding the benefits of AI, the majority of respondents (73.3% of females and 75.3% of males) expressed agreement that AI is beneficial. Agreement was highest among younger respondents aged ∼30 (100%) and lower among older respondents aged 51–60 (68%) (*p* = 0.040). There were no significant differences by gender, education level, years of practice, specialty, hospital type, or IT experience (Table 3).

In terms of trust in AI, around half of respondents expressed agreement overall. Agreement was highest among younger respondents aged ∼30 (89.3%) and lower among older respondents, which was statistically significant (*p* = 0.004). Agreement was also significantly lower among those with bachelor’s degrees (38.8%) compared to master’s (58.7%) or doctorate (49.1%) degrees (*p* = 0.032).

For acceptance of AI, agreement was expressed by 58.5% of females and 63.4% of males (*p* = 0.548). Acceptance was significantly higher among younger respondents aged ∼30 (92.9%) and lower among older respondents (*p* = 0.009). It was also higher among those with IT experience (80%) compared to no IT experience (57%) (*p* = 0.035). These findings were available in Supplementary Tables 2–S4.

### 3.7 The factors drive to use AI

**Figure 2:**
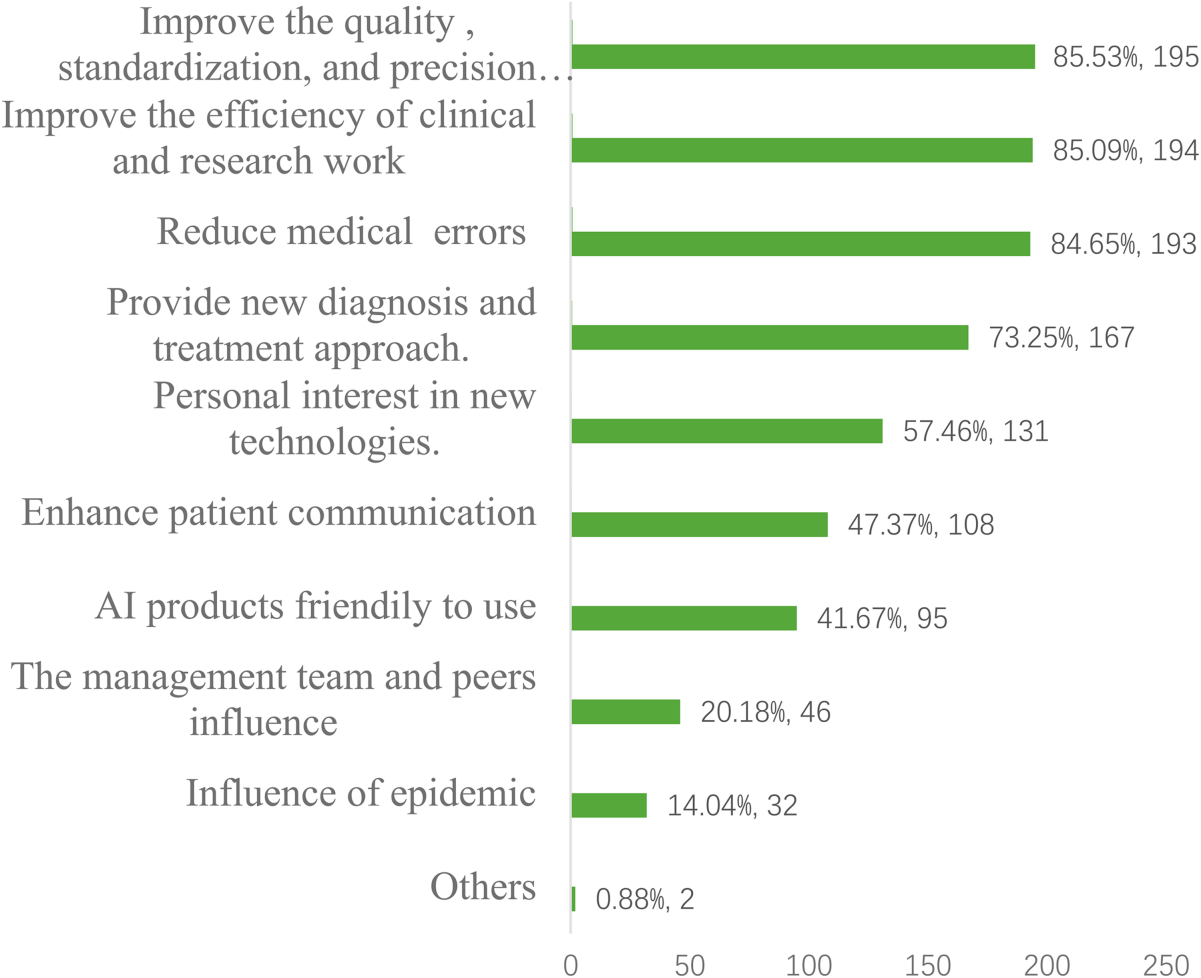
The factors drive to use AI.

The most selected factor was improving efficiency of clinical and research work, chosen by 194 respondents (85.09%). The second most common factor was improving quality, standardization, and precision medicine, selected by 195 respondents (85.53%). Reducing medical errors was selected by 193 respondents (84.65%), followed by providing new diagnosis and treatment approaches, selected by 167 respondents (73.25%). Personal interest in new technologies was a factor for 131 respondents (57.46%). Enhancing patient communication was selected by 108 respondents (47.37%). Influence from management and peers was a motivator for 46 respondents (20.18%). The COVID-19 pandemic was a factor for 32 respondents (14.04%).Only 2 respondents (0.88%) selected other motivational factors not already listed (Figure 2).

## 4 Discussion

To our knowledge, this study is the first online survey about Chinese oncologists’ attitudes towards AI. Our survey comprised a sample of 228 oncologists, their characters are basically consistent with the distribution of oncologists and oncologists in Chinese hospitals. 88.16% of oncologist in study exposure to AI technology, the demographic diversity allowed us to draw a comprehensive representation of oncologists’ understanding, perception, and acceptance of AI in their field.

The study revealed a moderate understanding of AI-related terms among oncologists. Less than half (44.17%) demonstrated high familiarity with terms like “artificial intelligence” and “machine learning,” and more less (34.21%) indicated understanding the limitation of AI. 35.09% hold neutral position. Other studies showed 23.8%–96% of healthcare professionals have a basic understanding of what AI is and definition (12–15). The Hah et al. found that AI knowledge and skills were not strongly correlated with diagnostic capability. In other words, having knowledge about AI did not necessarily improve an individual’s ability to diagnose conditions (16). This finding suggests that while many oncologists have some level of comprehension about AI, there is still a need for more extensive knowledge dissemination in the field.

Knowledge gaps were more pronounced among older, more experienced oncologists, indicating potential value in targeted educational initiatives. Younger individuals, those with higher education (particularly doctorate degrees), less clinical experience, and IT experience, are more likely to understand AI terms and limitations. As the understanding of AI becomes increasingly crucial in various fields, including medicine, these factors may influence the design of future educational initiatives and trainings. It might be beneficial to focus on enhancing AI literacy among older professionals, those with lower degrees, more clinical experience, and no IT experience.

Encouragingly, A majority of respondents (67.1%) expressed a willingness to learn about AI, and 67.98% of them perceive a need for oncologists to be trained on AI knowledge and applications. Respondents showed recognizing its growing relevance in medicine. Higher degrees of enthusiasm among younger oncologists also highlight the need to the results also reinforce the need for customized educational approaches based on baseline knowledge and attitudes towards AI among different demographic segments of healthcare professionals (17).

The majority of respondents expressed positive attitudes towards AI, with agreement that AI is beneficial (74.13%), trust in health AI technology (52.19%), acceptance of AI technology (61.53%), The systemic review showed that more than 60% of the respondents in 38 (84.44%) studies had an optimistic view to AI (18). which proved that AI is trusted and accepted by most doctors.

Nonetheless, the existence of a minority that expresses disagreement or strong disagreement is noteworthy. And belief that AI can exceed physician abilities in some areas nearly half (49.13%). This divergence indicates the presence of skepticism or resistance towards AI, underscoring the need for careful consideration and management of AI implementation in healthcare.

Notably, our study found younger and more highly educated clinicians tended to hold more favorable views of medical AI. Younger age was associated with greater agreement that AI is beneficial and higher acceptance of AI technology. This resonates with findings that younger generations tend to have more positive views of emerging technologies like AI, and gender did not show the difference (19).

However, another study in UK indicated that the younger population, aged 16–24, who are less inclined to think that AI will lead to an improvement in the quality of care (20). Perhaps this skepticism might stem from their concerns regarding AI.

Higher education level also corresponded with increased trust in AI. This aligns with literature suggesting greater AI knowledge shapes more favorable attitudes (21).Prior IT experience related to stronger agreement that AI can exceed physician abilities and higher acceptance of AI. Hands-on technology exposure may improve AI perceptions. University hospital affiliation vs. non-university hospitals did not significantly impact AI perspectives. This contrasts past studies showing academic settings relate to more positive AI views.

The study also showed that there is a high level of agreement among respondents that AI will bring innovation to oncology, the healthcare system, and healthcare in general. Most respondents have an optimistic view of the role AI can play in advancing these fields. This suggests that respondents not only see the benefits of AI in current applications but also believe in its future potential to drive the healthcare sector’s evolution. The low standard deviations show this is a consistent opinion across the sample.

“Some aspects of current AI technology have exceeded the average ability of doctors,” the largest portion of respondents (30.26%) chose neutral, 53.07% agree and strongly agree the statement. with a standard deviation of 1.21 and a variance of 1.46.it can be concluded that there is a mixed opinion regarding whether some aspects of current AI technology have exceeded the average ability of doctors. The largest portion of respondents chose neutral, indicating uncertainty or lack of consensus on this matter.

Regarding the statement “AI will not replace doctors,” the majority of respondents (53.95%) strongly agree, and 24.56% agree. These results suggest a strong consensus among the respondents that AI will not replace human doctors in the future with a standard deviation of 1.1 and a variance of 1.2. This indicated that people believe doctors still maintain an advantage in abilities over AI in some respects currently, but AI may complement doctors in the future rather than replace them outright (22–24).

The majority of respondents expressed an openness and willingness to use AI in healthcare if given the opportunity. The level of willingness was moderately high for both statements, though slightly higher for general openness to using AI. Some variability in the responses suggests there are still some reservations or uncertainty about the extent of AI use among some people. But overall, the results imply that most would welcome AI applications in their healthcare.

the factors driving doctors’ use of AI in healthcare reflect a holistic approach that encompasses patient care, innovation, efficiency, and quality improvement. The findings suggest that doctors are receptive to AI technologies that can enhance their practice, improve patient outcomes, and advance the field of healthcare. The results indicate doctors are driven to use AI largely for its potential benefits to healthcare delivery and outcomes. The view seems to be AI complementing and supporting, not replacing, human roles. Concerns of AI automating doctors out of jobs do not appear to be borne out by these motivational factors.

The respondents readiness to adopt new technologies was also evident, with a mean score of 4.1 for the statement “I'm usually willing to try new technologies”. Similarly, a high score was observed for the statement “I prefer to use high technology than people around me”. These findings, in conjunction with the interest in learning about AI, suggest that the integration of AI in oncology could be well-received, provided the appropriate training and resources are made available.

From the oncologists’ perspective, the top factors driving doctors’ use of AI in healthcare reflect a holistic approach that encompasses patient care, innovation, efficiency, and quality improvement. The findings suggest that doctors are willing to AI technologies that can enhance their practice, improve patient outcomes, and advance the field of healthcare. The view seems to be AI complementing and supporting, not replacing, human roles.

In addition, for the safe and effective application of AI integrate into healthcare. China is building regulatory framework for AI, which includes legislation, policy formulation, safety assessment, data protection, ethical considerations, and ongoing supervision.

In 2017, The State Council issued “New Generation Artificial Intelligence Development Plan” to guide AI development (25). The “Data Security Law” and the “Personal Information Protection Law” implemented in 2021 to require AI companies to anonymize data to protect patient privacy (26, 27).The National Medical Products Administration (NMPA) is responsible for conducting rigorous clinical trials and pre-market approvals for medical AI products (28). Some medical societies have established ethical review and transparency requirements to ensure that AI technologies meet ethical standards (29). This comprehensive series of regulatory measures aims to ensure that health artificial intelligence technology can be safely and effectively integrated into China’s healthcare system.

## 5 Limitation

As with all research, this study is not without limitations, and its results should be interpreted with caution. First, the study’s relatively small sample size of oncologists potentially limits the broader applicability of the findings across the entire oncologist population. Second, the data was collected through self-reported measures, which could introduce bias, as those with a pre-existing interest or positive outlook on AI might have been more likely to participate. Furthermore, the responses might not accurately reflect the reality due to subjectivity in self-assessment. Lastly, the study’s results are based on experiences with the respondent specific AI products, which may not be representative of other categories of AI technologies. As a result, the findings might not extend universally to all types of AI applications in oncology.

## 6 Conclusion

Our study finds that oncologists moderately understand AI-related terms and limitations, expressing a keen interest in deepening their AI knowledge. They perceive AI as beneficial to medicine, but their acceptance is tempered with caution about AI’s potential to replace doctors. While they express a willingness to utilize AI if available and acknowledge AI’s potential to innovate oncology and healthcare, their acceptance of current AI technologies remains cautiously optimistic. These results suggest a significant willingness among oncologists to enhance their AI knowledge. Younger oncologists with advanced degrees and IT experience demonstrate greater openness to and understanding of AI. The study underlines the importance of developing AI curricula for physicians. Training for oncologists with different characteristics is necessary to help implement AI.

## Data Availability

The datasets presented in this article are not readily available because the raw data supporting the conclusions of this article will be made available by the authors, without undue reservation. Requests to access the datasets should be directed to xiaomin0335@cqu.edu.cn.
