# Supplementary material for: Attitudes and perceptions of Chinese oncologists towards artificial intelligence in healthcare: a cross-sectional survey
Source: Front Digit Health. 2024 Sep 3;6:1371302. doi: 10.3389/fdgth.2024.1371302 (PMC11405309; doi:10.3389/fdgth.2024.1371302)
Supplement: Supplementary file 1 [file Datasheet1.pdf]

SUPPLMENTAL TABLE

Table 1：Demographics（n=228）

Gender			
	Male	135	59.21%
	Female	93	40.79%
Age			
	~30	28	12.28%
	31~40	95	41.67%
	41~50	80	35.09%
	51~60	25	10.96%
Years of Clinical Practice			
	0~10	49	21.49%
	11~20	126	55.26%
	21~	53	23.25%
Education Degree			
	Bachelor	89	39.04%
	Master	83	36.40%
	Doctor	56	24.56%
Specialty			
	Medical oncology.	97	42.54%
	Surgical oncology	77	33.77%
	Radiation therapy	40	17.54%
	Others	14	6.14%
Hospital Type			
	University Hospital	148	64.91%
	Non-University Hospital	80	35.09%
Experience with IT Project			
	Yes	35	15.35%
	No	193	84.65%

Table 2: I believe "AI is beneficial." (n=228)

Gender		No.	Strongly Disagree/ Disagree	Neutral	Strongly Agree/ Agree	X^2	P
	Male	135	7(5.2%)	29(21.5%)	99(73.3%)	0.152	0.927
	Female	93	5(5.4%)	18(19.4%)	70(75.3%)		
Age							
	~30	28	0(0%)	0(0%)	28(100%)	13.175	0.040*
	31~40	95	4(4.2%)	25(26.3%)	66(69.5%)		
	41~50	80	6(7.5%)	16(20%)	58(72.5%)		
	51~60	25	2(8%)	6(24%)	17(68%)		
Education Degree							
	Bachelor	49	5(10.2%)	14(28.6%)	30(61.2%)	6.319	0.177
	Master	126	5(4%)	24(19%)	97(77%)		
	Doctor	53	2(3.8%)	9(17%)	42(79.2%)		
Year of clinical Practice							
	~10	89	1(1.1%)	18(20.2%)	70(78.7%)	5.397	0.249
	11~20	83	6(7.2%)	17(20.5%)	60(72.3%)		
	20~	56	5(8.9%)	12(21.4%)	39(69.6%)		
Specialty							
	Medical oncology.	97	6(6.2%)	21(21.6%)	70(72.2%)	1.293	0.972
	Surgical oncology	77	4(5.2%)	16(20.8%)	57(74%)		
	Radiation therapy	40	2(5%)	7(17.5%)	31(77.5%)		
	Others	14	0(0%)	3(21.4%)	11(78.6%)		
Hospital Type							
	University Hospital	148	6(4.1%)	25(16.9%)	117(79.1%)	5.39	0.068
	Non University Hospital	80	6(7.5%)	22(27.5%)	52(65%)		
IT Experience							
	Yes	35	0(0%)	5(14.3%)	30(85.7%)	3.729	0.155
	No	193	12(6.2%)	42(21.8%)	139(72%)		

Table 3: I Trust in health AI technology. (n=228)

			Strongly Disagree/ Disagree	Neutral	Strongly Agree/ Agree	X^2	P
Gender							
	Male	135	15(11.1%)	52(38.5%)	68(50.4%)	0.47	0.791
	Female	93	10(10.8%)	32(34.4%)	51(54.8%)		
Age							
	~30	28	1(3.6%)	2(7.1%)	25(89.3%)	18.899	0.004*
	31~40	95	11(11.6%)	42(44.2%)	42(44.2%)		
	41~50	80	9(11.2%)	31(38.8%)	40(50%)		
	51~60	25	4(16%)	9(36%)	12(48%)		
Education Degree							
	Bachelor	49	10(20.4%)	20(40.8%)	19(38.8%)	10.529	0.032*
	Master	126	12(9.5%)	40(31.7%)	74(58.7%)		
	Doctor	53	3(5.7%)	24(45.3%)	26(49.1%)		
Year of clinical Practice							
	~10	89	6(6.7%)	29(32.6%)	54(60.7%)	6.015	0.198
	11~20	83	10(12%)	35(42.2%)	38(45.8%)		
	20~	56	9(16.1%)	20(35.7%)	27(48.2%)		
Specialty							
	Medical oncology.	97	11(11.3%)	36(37.1%)	50(51.5%)	3.504	0.744
	Surgical oncology	77	8(10.4%)	31(40.3%)	38(49.4%)		
	Radiation therapy	40	6(15%)	12(30%)	22(55%)		
	Others	14	0(0%)	5(35.7%)	9(64.3%)		
Hospital Type							
	University Hospital	148	16(10.8%)	52(35.1%)	80(54.1%)	0.623	0.732
	Non University Hospital	80	9(11.2%)	32(40%)	39(48.8%)		
IT Experience							
	Yes	35	2(5.7%)	10(28.6%)	23(65.7%)	3.256	0.196
	No	193	23(11.9%)	74(38.3%)	96(49.7%)		

Table 4: I accept AI technology. (n=228)

Gender			Strongly Disagree/ Disagree	Neutral	Strongly Agree/ Agree	X²	P
	Male	135	14(10.4%)	42(31.1%)	79(58.5%)	1.203	0.548
	Female	93	6(6.5%)	28(30.1%)	59(63.4%)		
Age							
	~30	28	1(3.6%)	1(3.6%)	26(92.9%)	17.134	0.009*
	31~40	95	6(6.3%)	35(36.8%)	54(56.8%)		
	41~50	80	9(11.2%)	26(32.5%)	45(56.2%)		
	51~60	25	4(16.0%)	8(32%)	13(52%)		
Education Degree							
	Bachelor	49	8(16.3%)	17(34.7%)	24(49%)	6.574	0.16
	Master	126	10(7.9%)	37(29.4%)	79(62.7%)		
	Doctor	53	2(3.8%)	16(30.2%)	35(66%)		
Year of clinical Practice							
	~10	89	2(2.2%)	26(29.2%)	61(68.5%)	10.773	0.029*
	11~20	83	10(12.0%)	23(27.7%)	50(60.2%)		
	20~	56	8(14.3%)	21(37.5%)	27(48.2%)		
Specialty							
	Medical oncology.	97	8(8.2%)	31(32%)	58(59.8%)	1.945	0.925
	Surgical oncology	77	8(10.4%)	23(29.9%)	46(59.7%)		
	Radiation therapy	40	4(10.0%)	12(30%)	24(60%)		
	Others	14	0(0.0%)	4(28.6%)	10(71.4%)		
Hospital Type							
	University Hospital	148	12(8.1%)	43(29.1%)	93(62.8%)	0.957	0.62
	Non-University Hospital	80	8(10.0%)	27(33.8%)	45(56.2%)		
IT Experience							
	Yes	35	1(2.9%)	6(17.1%)	28(80%)	6.716	0.035*
	No	193	19(9.8%)	64(33.2%)	110(57%)		
